# The effect of SportsMetrics training program on functional performance, landing error, dynamic knee valgus and hamstrings/quadriceps strength in athletes: a systematic review and meta-analysis

**DOI:** 10.1186/s13102-026-01983-4

**Published:** 2026-08-14

**Authors:** Fereshteh Kazemi pakdel, Ahmad Kazemi pakdel, Elham Hajihoseini, Mohamad Mottaghitalab, Hamed Zarei

**Affiliations:** 1 Corrective Exercises and Sports Injury Department, Faculty of Physical Education & Sport Sciences, University of Guilan, Rasht, 4199613776 Iran; 2 Department of Sports Injury and Corrective Movement, Faculty of Physical Education, University of Guilan, Rasht, Iran; 3 Sports Biomechanics Department, Faculty of Physical Education & Sport Sciences, University of Guilan, Rasht, 4199613776 Iran

**Keywords:** Neuromuscular training, Functional performance, ACL prevention, Athletes

## Abstract

**Objective:**

The SportsMetrics training (SMT) program is a comprehensive injury prevention initiative designed for athletes, focusing on improving strength, flexibility, and overall performance through targeted exercises and assessments. Therefore, the purpose of this systematic review is to examine the effect of SMT program on functional performance, landing error, dynamic knee valgus and hamstrings/quadriceps strength ratio in athletes.

**Methods:**

Primary sources were sourced from four databases: SCOPUS, PubMed, CENTRAL, and Web of Science. The search period spanned from the inception of these databases up to March 29, 2025. The inclusion criteria were defined as follows: (1) Population: Professional or recreational athletes; (2) Intervention: SMT program; (3) Comparator: Comparison of SMT program with either a non-training control group or conventional training (CT); (4) Outcomes: functional performance, landing error, dynamic knee valgus and muscle strength; (5) Study Designs: Randomized controlled trials; (6) Peer-reviewed articles published in English. Standardized mean differences (SMDs), and 95% confidence intervals (CIs) were computed using either random or fixed-effect models for the outcomes.

**Results:**

After the screening process, a total of 20 studies comprising 825 participants were included in the systematic review. The results of the meta-analysis showed that SMT improved agility (1.24 [95% CI: 0.53–1.95], *p* = 0.001), core endurance (1.16 [95% CI: 0.43–1.89], *p* = 0.001), dynamic balance (1.29 [95% CI: 0.92–1.66], *p* = 0.001), jump height (0.44 [95% CI: 0.12–0.77], *p* = 0.007), power (1.02 [95% CI: 0.44–1.60], *p* = 0.001), speed (0.57 [95% CI: 0.28–0.87], *p* = 0.001), hamstring strength (1.29 [95% CI: 1.20–1.38], *p* = 0.001), quadriceps strength (2.43 [95% CI: 2.32–2.54], *p* = 0.001), and hamstrings/quadriceps strength ratio (1.22 [95% CI: 1.13–1.31], *p* = 0.001). Also, results showed that SMT reduced in the knee valgus (3.46 [95% CI: 2.10–4.81], *p* = 0.001), and landing errors (2.45 [95% CI: 2.06–2.78], *p* = 0.001).

**Conclusion:**

The present meta-analysis demonstrates that the SMT program significantly improved functional performance, increased hamstrings/quadriceps strength, and reduced landing errors and dynamic knee valgus in athletes. Despite the robust evidence, there remains a gap in original research directly linking SMT to injury rates, underscoring the need for future studies to explore this relationship.

**Trial registration:**

CRD420251020418.

**Supplementary Information:**

The online version contains supplementary material available at https://doi.org/10.1186/s13102-026-01983-4.

## Introduction

The anterior cruciate ligament (ACL) tear is recognized as one of the most serious and complex injuries affecting the knee [1]. Research indicates that female athletes experience ACL injuries at a rate 2 to 8 times higher than their male counterparts [2]. Notably, there are differences in ACL injury rates between genders across different sports [3]. For instance, the reported injury rates for females, expressed as the number of injuries per 1,000 athlete-exposures (where one exposure equals one game or practice), are 0.28 for soccer, 0.23 for basketball, 0.09 for volleyball, and the highest at 0.33 for gymnastics [4]. Such injuries can result in diminished athletic performance, prolonged absences from training, knee joint laxity, impaired proprioception, and an increased risk of early-onset osteoarthritis [5]. Additionally, the financial burden associated with post-traumatic care, including surgical costs, rehabilitation, and psychological impacts, underscores the importance of implementing injury prevention programs [6].

In recent years, injury prevention has emerged as a significant concern [7, 8]. Prevention protocols can be broadly categorized into two main types: neuromuscular (NM) training and specialized warm-up routines [9]. NM training is recognized as the most effective method for preventing injuries and improving performance techniques [10]. Research indicates that multi-component prevention programs yield better outcomes in ACL injury prevention compared to single-component approaches [11]. Within this framework, the SportsMetrics training (SMT) protocol is specifically designed to prevent injuries and enhance athletic performance [12].

The SMT program is a comprehensive ACL injury prevention initiative tailored for female athletes [13]. It incorporates various training elements customized for specific sports, focusing on improving landing techniques, optimizing lower extremity alignment, enhancing neuromuscular control, and increasing range of motion during landings [14]. These enhancements aim to reduce knee valgus (KV) and improve overall athletic performance [15]. Each session begins with a specific warm-up, followed by a series of exercises that include plyometric, endurance, speed, strength, agility, and reaction training, concluding with a cool-down period [16].

The relationship between agility, speed, power, muscle strength (specifically quadriceps and hamstrings), core stability, and dynamic balance is intricately linked to the risk of ACL injuries. Agility relies heavily on core stability and dynamic balance, as a stable core facilitates proper posture and alignment during movement, enhancing an athlete’s ability to perform quick directional changes without compromising knee integrity [17]. Speed, essential for competitive performance, is influenced by these factors; athletes who can accelerate and decelerate effectively while maintaining balance are less likely to experience the awkward landings or sudden stops that can precipitate ACL injuries [17]. Power underpins both agility and speed, and inadequate power can lead to inefficient movement patterns that increase injury risk [17]. The strength of the quadriceps and hamstrings is particularly relevant, as these muscle groups work together to stabilize the knee joint during dynamic activities. A muscular imbalance, where the quadriceps are significantly stronger than the hamstrings, can predispose athletes to ACL injuries, as the hamstrings play a protective role in controlling knee motion during high-impact activities [18]. Additionally, dynamic balance integrates all these variables; athletes with superior dynamic balance can maintain stability and control during complex movements, reducing the likelihood of falls or improper landings that could lead to ACL ruptures [15]. Therefore, a holistic training approach that addresses all these factors is essential for optimizing athletic performance while minimizing the risk of injury.

Numerous meta-analyses evaluating the efficacy of NM training programs in lowering ACL injury rates have concluded that these interventions are generally effective [19–23]. However, these analyses aggregated data from multiple studies [19, 20, 23]. In contrast, systematic reviews that did not pool data from various programs have indicated that while some training programs are effective [22], others do not significantly decrease the risk of ACL injuries [21]. These findings emphasize the importance of recognizing the variations in training protocols and study designs, as they are crucial for understanding the differences in outcomes.

Moreover, while research has explored the relationship between sex-specific anatomical differences and ACL injuries, these differences are largely non-modifiable [24]. NM control of knee joint stability has been highlighted as a significant risk factor, particularly because differences in movement and muscle activation patterns between genders are believed to contribute to a heightened risk of ACL injuries in females [25]. However, the effectiveness of injury prevention programs that include elements of NM training, such as SMT, varies significantly. Therefore, the aim of this research is to perform a systematic review and meta-analysis to examine the impact of SMT on the functional performance, landing error, dynamic knee valgus and hamstrings/quadriceps strength ratio in athletes. The difference is that the current systematic review focused on functional metrics, while those systematic reviews focused on injury rates; Therefore, this systematic review will identify the specific variables that have been studied and present the results of these studies in a meta-analytic format.

## Method

This systematic review adhered to the protocols established by the Preferred Reporting Items for Systematic Reviews and Meta-Analyses (PRISMA) [26] (refer to supplementary files 1 and 2). The review protocol has been registered in PROSPERO with the identification number CRD420251020418.

### Search strategy

Primary sources were gathered from four databases: PubMed, SCOPUS, CENTRAL (Cochrane Central Register of Controlled Trials), and Web of Science. The search period spanned from the inception of these databases up to March 29, 2025 (no restrictions were placed on the publication date). Initially, Medical Subject Headings (MeSH) terms were utilized to inform the selection of keywords, which were later refined to ensure comprehensive coverage of relevant studies. Additionally, Google Scholar was utilized to broaden our search, incorporating articles from various databases. Following the selection process, we examined the references of the included studies to identify any potentially overlooked citations. The electronic databases were searched using combinations of the following groups of keywords: (1) “knee injury prevention” OR “knee injury” OR “anterior cruciate ligament injury prevention” OR “injury prevention” OR “prevention” OR “anterior cruciate ligament prevention " OR “ACL prevention” OR “knee prevention” OR “prospective knee prevention”; AND (2) " SportsMetrics program” OR “injury prevention program” OR “neuromuscular training” OR “neuromuscular intervention” OR " SportsMetrics training” OR SportsMetrics; (3) athletes OR “sport player” OR player OR sport OR " recreational player” OR " recreational sport”; (4) “Performance metrics” OR “Functional testing” OR “Performance optimization” OR “Performance profiling” OR “Performance standards” OR Function OR Performance. The “AND” operator was used to connect the three groups of keywords, while the “OR” operator was employed within each individual keyword group.

### Eligibility criteria

The inclusion criteria were defined as follows: (1) Population: Professional or recreational athletes; (2) Intervention: SMT program; (3) Comparator: Comparison of SMT program with either a non-training control group or conventional training (CT); (4) Outcomes: functional performance, landing error, dynamic knee valgus and muscle strength; (5) Study Designs: Randomized controlled trials; (6) Peer-reviewed articles published in English. Two independent researchers (F.KP. and H.Z.) performed the search and screened titles and abstracts according to the predetermined criteria. Any discrepancies were addressed through discussion. The level of agreement between the researchers was evaluated using Cohen’s kappa (κ). A κ value of ≤ 0.4 indicated poor agreement, 0.41–0.6 represented moderate agreement, 0.61–0.8 reflected good agreement, and 0.81–1 signified excellent agreement [27]. 

### Data extraction

Researchers (F.KP. and H.Z.) independently gathered data from the studies utilizing several metrics, including the first author’s name, study design, participant characteristics (such as sample size, age range or mean age with standard deviation (SD), and sex distribution), outcomes measure, measurement methods and units, along with information regarding the training programs and their specific attributes (refer to Table 1).

**Table 1:** General description of the samples included in the individual studies

Source, year	Study design	Sample size	Sex	Age [years]	Groups	Participants characteristics	Intervention	Training characteristics= Frequency (time a week)/ Duration (week)	Outcomes measure
Baldon et al. 2014 [28]	RCT, Two arms	36	F	EXP = 20.11 ± 1.68 CON = 20.17 ± 1.54	EXP = 18 CON = 18	Recreational female athletes	EXP= SportsMetrics training CON= Routine training	3/8	- Hamstrings peak torque (N·m) - Quadriceps peak torque (N·m) - Hamstrings/quadriceps (Ratios)
Fakhraei Rad et al. 2024 [29]	RCT, Two arms	84	F	EXP (A) = 20.40 ± 1.90 CON (A) = 21.30 ± 2.50 EXP (B) = 20.40 ± 1.90 CON (B) = 21.30 ± 2.50 EXP (C) = 20.40 ± 1.90 CON (C) = 21.30 ± 2.50	EXP (A) = 15 CON (A) = 15 EXP (B) = 15 CON (B) = 15 EXP (C) = 12 CON (C) = 12	Females’ athletes with dynamic knee valgus (volleyball, basketball and soccer players)	EXP= SportsMetrics training CON= Routine training	3/6	- LESS (score)
Wilkerson et al. 2004 [30]	RCT, Two arms	19	F	EXP = 19 ± 1.4 CON = 19 ± 1.1	EXP = 11 CON = 8	Female collegiate basketball players	EXP= SportsMetrics training CON= Routine training	3/6	- Agility (sec) - Hamstrings peak torque (N·m) - Quadriceps peak torque (N·m) - Hamstrings/quadriceps (Ratios)
Grandstrand et al. 2006 [31]	RCT, Two arms	24	F	EXP = 10.2 ± 2.3 CON = 9.8 ± 1.5	EXP = 12 CON = 12	Female soccer players	EXP= SportsMetrics training CON= Routine training	2/8	- Jump height (cm)
Kalantariyan et al. 2025 [32]	RCT, Two arms	30	F	EXP = 11.1 ± 1.4 CON = 10.8 ± 1.3	EXP = 15 CON = 15	Young Futsal Players	EXP= SportsMetrics training CON= Routine training	3/6	- Dynamic balance (cm) - Agility (sec) - Power (cm) - Speed (sec)
Mohammadi et al. 2022.a [33]	RCT, Two arms	24	F	EXP = 20.2 ± 8.02 CON = 19.75 ± 1.71	EXP = 12 CON = 12	Female soccer players with dynamic knee valgus	EXP= SportsMetrics training CON= Routine training	3/6	- Agility (sec) - Power (cm) - Speed (sec) - Knee valgus (angle)
Mohammadi et al. 2022.b [34]	RCT, Two arms	30	F	EXP = 20.4 ± 1.9 CON = 21.3 ± 2.5	EXP = 15 CON = 15	Female Volleball players with dynamic knee valgus	EXP= SportsMetrics training CON= Routine training	3/6	- LESS (score) - Knee valgus (angle)
Mohammadi et al. 2022.c [35]	RCT, Two arms	24	F	EXP = 20.08 ± 2.02 CON = 19.1 ± 1.75	EXP = 12 CON = 12	Female soccer players with dynamic knee valgus	EXP= SportsMetrics training CON= Routine training	3/6	- LESS (score) - Knee valgus (angle)
Mohammadi et al. 2023 [15]	RCT, Six arms	84	F	EXP (A) = 20.40 ± 1.90 CON (A) = 21.30 ± 2.50 EXP (B) = 20.40 ± 1.90 CON (B) = 21.30 ± 2.50 EXP (C) = 20.40 ± 1.90 CON (C) = 21.30 ± 2.50	EXP (A) = 15 CON (A) = 15 EXP (B) = 15 CON (B) = 15 EXP (C) = 12 CON (C) = 12	Females’ athletes with dynamic knee valgus (volleyball, basketball and soccer players)	EXP= SportsMetrics training CON= Routine training	3/6	- Dynamic balance (cm) - Agility (sec) - Jump height (cm) - Speed (sec) - Knee valgus (angle) - Core endurance (sec)
Mohammadi et al. 2024.a [36]	RCT, Two arms	24	F	EXP = 20.1 ± 2.0 CON = 19.7 ± 1.7	EXP = 12 CON = 12	Female soccer players with dynamic knee valgus	EXP= SportsMetrics training CON= Routine training	3/6	- Core endurance (sec) - Dynamic balance (cm) - Knee valgus (angle)
Mohammadi et al. 2024.b [37]	RCT, Two arms	30	F	EXP = 21.30 ± 2.50 CON = 20.40 ± 1.90	EXP = 15 CON = 15	Female Volleyball Athletes with Neuromuscular Knee Valgus Defect	EXP= SportsMetrics training CON= Routine training	3/8	- Dynamic balance (cm) - Agility (sec) - Power (cm) - Speed (sec) - Knee valgus (angle) - Core endurance (sec)
Noyes et al. 2015 [38]	RCT, Two arms	212	F	EXP = 13–18 CON = 13–18	EXP = 100 CON = 112	Female Adolescent Athletes	EXP= SportsMetrics training CON= Routine training	3/6	- Hamstrings peak torque (N·m) - Quadriceps peak torque (N·m) - Hamstrings/quadriceps (Ratios)
Roso-Moliner et al. 2023 [39]	RCT, Two arms	38	F	EXP = NR CON = NR	EXP = 18 CON = 20	Female football players	EXP= SportsMetrics training CON= Routine training	1/10	- Agility (sec) - Jump height (cm) - Speed (sec)
Saki et al. 2020 [13]	RCT, Two arms	22	F	EXP = 18.40 ± 0.5 CON = 18.64 ± 1.08	EXP = 11 CON = 11	Female football players	EXP= SportsMetrics training CON= Routine training	3/6	- Agility (sec) - Power (cm) - Speed (sec)
Saki et al. 2021 [40]	RCT, Two arms	29	F	EXP = 18.40 ± 0.5 CON = 18.64 ± 1.08	EXP = 14 CON = 15	Female football players	EXP= SportsMetrics training CON= Routine training	3/6	- LESS (score) - Dynamic balance (cm)
Saki et al. 2023 [41]	RCT, Two arms	29	F	EXP = 18.40 ± 0.5 CON = 18.64 ± 1.08	EXP = 14 CON = 15	Female football players	EXP= SportsMetrics training CON= Routine training	3/6	- Tuck jump (score) - Core endurance (sec)
Shahrjerdi et al. 2021 [42]	RCT, Two arms	25	F	EXP = 15.92 ± 1.18 CON = 17.75 ± 2.63	EXP = 13 CON = 12	Female young volleyball players	EXP= SportsMetrics training CON= Routine training	3/6	- LESS (score)
Singh et al. 2015 [43]	RCT, Two arms	40	F	EXP = 20.0 ± 1.2 CON = 20.0 ± 1.2	EXP = 20 CON = 20	Female basketball elite players	EXP= SportsMetrics training CON= Routine training	3/6	- Jump height (cm) - Agility (sec) - Knee valgus (angle)
Tsang et al. 2011 [44]	RCT, Two arms	25	F	EXP = 20.32 ± 1.92 CON = 20.32 ± 1.92	EXP = 11 CON = 14	Female athletes	EXP= SportsMetrics training CON= Routine training	3/6	- Hamstrings peak torque (N·m) - Quadriceps peak torque (N·m) - Hamstrings/quadriceps (Ratios)
Vescovi et al. 2008 [45]	RCT, Two arms	18	F	EXP = 20.3 ± 1.2 CON = 19.9 ± 1.6	EXP = 10 CON = 8	Recreational basketball players	EXP= SportsMetrics training CON= Routine training	3/6	- Jump height (cm) - Power (cm) - Speed (sec)

*NR* Not reported, *SD* Standard deviation, *M* Male, *F* Female, *EXP* Experimental group, *CON* Control group, *RCT* Randomized control trial, *LESS* Landing error scoring system

### Quality of evidence

Quality assessment scores for the eligible studies were evaluated using the Physiotherapy Evidence Database (PEDro). The total PEDro score, which ranges from 0 to 11, considers factors such as the reporting of statistical analysis and criteria for assessing internal validity. Studies that received scores between 7 and 11 were categorized as having “high” methodological quality, those with scores of 5 to 6 were deemed “fair,” and studies scoring ≤ 4 were classified as “poor” [46] (refer to Table 2 for additional details).

**Table 2:** PEDro scale ratings

Study	Eligibility Criteria	Random Allocation	Concealed Allocation	Groups Similar At Baseline	Blind Subject	Blind Therapist	Blind Assessor	Follow-Up	Intention-to-Treat Analysis	Between-Group Comparisons	Point Measures and Variability	PEDro Score
Baldon et al. 2014 [28]	1	1	0	1	1	0	0	0	1	1	0	6.11
Fakhraei Rad et al. 2024 [29]	1	1	1	1	1	0	0	0	1	1	1	8.11
Wilkerson et al. 2004 [30]	1	0	0	1	1	0	0	0	1	1	0	5.11
Grandstrand et al. 2006 [31]	1	0	1	1	1	1	0	0	1	1	0	7.11
Kalantariyan et al. 2025 [32]	1	1	1	1	1	0	0	0	1	1	1	8.11
Mohammadi et al. 2022.a [33]	1	1	0	1	1	0	0	0	1	1	1	7.11
Mohammadi et al. 2022.b [34]	1	1	0	1	1	0	0	0	1	1	1	7.11
Mohammadi et al. 2022.c [35]	1	1	0	1	1	0	0	0	1	1	1	7.11
Mohammadi et al. 2023 [15]	1	1	0	1	1	0	0	0	1	1	1	7.11
Mohammadi et al. 2024.a [36]	1	1	0	1	1	0	0	0	1	1	1	7.11
Mohammadi et al. 2024.b [37]	1	1	0	1	1	0	0	0	1	1	1	7.11
Noyes et al. 2015 [38]	1	1	0	1	1	0	0	0	1	1	1	7.11
Roso-Moliner et al. 2023 [39]	1	1	1	1	1	0	0	0	1	1	1	8.11
Saki et al. 2020 [13]	1	1	0	1	1	1	0	0	1	1	0	7.11
Saki et al. 2021 [40]	1	1	0	1	1	1	0	0	1	1	0	7.11
Saki et al. 2023 [41]	1	1	0	1	1	1	0	0	1	1	0	7.11
Shahrjerdi et al. 2021 [42]	1	1	0	1	1	0	0	0	1	1	0	6.11
Singh et al. 2015 [43]	1	1	0	1	1	0	0	0	1	1	0	6.11
Tsang et al. 2011 [44]	1	1	0	1	1	0	0	0	1	1	0	6.11
Vescovi et al. 2008 [45]	1	1	0	1	1	0	0	0	1	1	0	6.11

### Statistical analyses

Heterogeneity was assessed using the I² index, with the following thresholds: 0%–30% indicating no heterogeneity; 30%–50% representing low heterogeneity; 50%–75% denoting moderate heterogeneity; and 75%–100% indicating high heterogeneity. In this study, both random and fixed-effects models were utilized to manage between-study heterogeneity and sampling error. A random effects model was applied when I² exceeded 50% [47]. Standardized mean differences (SMDs), and 95% confidence intervals (CIs) were computed using either random or fixed-effect models for the outcomes, with a significance level set at *p* ≤ 0.05. Funnel plots and Egger’s tests were visually examined to identify publication biases, with Egger’s test serving as a secondary measure when *p* < 0.1. The trim-and-fill correction method was employed as needed to address potential publication biases [48]. Statistical analysis was performed using Comprehensive Meta-Analysis version 2.0 (Biostat Inc, Englewood, New Jersey).

## Results

**Fig. 1:**
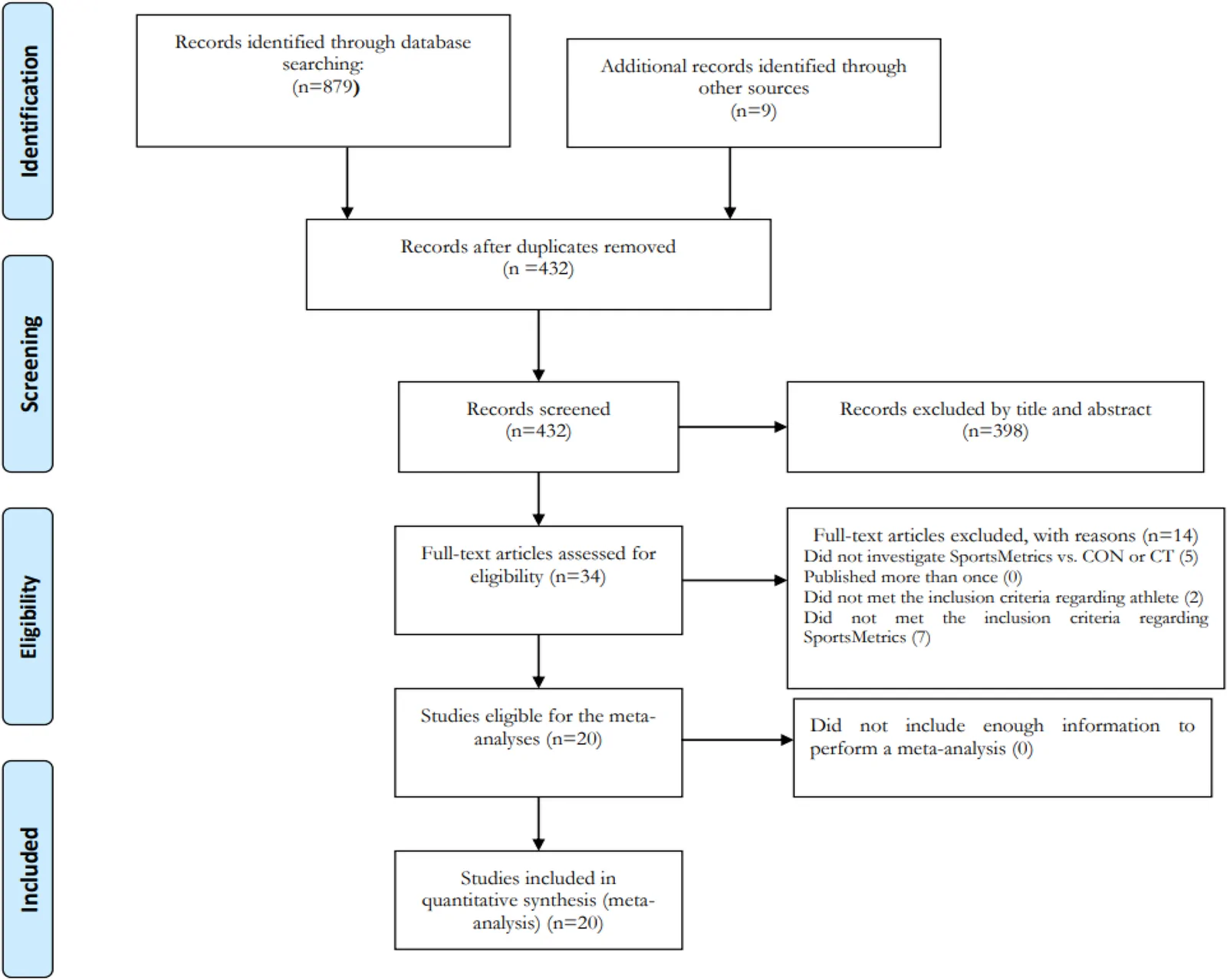
Flow diagram of systematic literature search. CON: control; CT: conventional training

A total of 879 potentially eligible studies were gathered from four databases. Furthermore, 9 additional records were identified by screening reference lists through the ancestry method. After excluding 456 duplicate studies, 432 relevant titles and abstracts were assessed, leading to the exclusion of another 398 abstracts. This process resulted in 34 full-text studies being selected for comprehensive evaluation. Of these, 14 studies were excluded for failing to meet the eligibility criteria. In the end, 20 studies, comprising a total of 825 participants, were included in the systematic review (Fig. 1).

### Quality of evidence

According to the PEDro Scale, among the 20 studies examining the effects of SMT program on functional outcomes of athletes, 14 studies (70%) received high scores (ranging from 7 to 11), while the remaining 6 studies (30%) were classified within the fair score range (5 to 6). Overall, the studies included in this review demonstrated a satisfactory quality as assessed by the PEDro Scale, indicating their reliability for the current meta-analysis (refer to Table 2).

### Quality of the evidence using the GRADE system

The quality of evidence was assessed utilizing the methodology established by the GRADE system [49]. This framework identifies five factors that can adversely affect the quality of evidence derived from a trial: study design, risk of bias, inconsistency, indirectness, and imprecision. Each of these factors was evaluated and classified as follows: not serious (no point reduction), serious (1-point reduction), or very serious (2-point reduction), with scores assigned based on the risk of bias in the study. An evaluation of the quality of studies using the GRADE system indicates that all assessed outcomes are of high to moderate quality. Consequently, the conclusions drawn from the evidence in this study can be made with a high degree of confidence. (refer to supplementary file 3).

### Outcomes characteristics

Among the 20 studies included in the systematic review, eight intervention arms investigated the impact of the SMT program on agility [13, 15, 30, 32, 33, 37, 39, 43]. In agility assessments, time is a critical factor; shorter completion times indicate better performance. These tests evaluate an individual’s capacity to change direction swiftly and efficiently while maintaining control and balance, and are commonly utilized in sports, fitness evaluations, and rehabilitation contexts. In addition to, five intervention arms also investigated the impact of the SMT program on jump height [15, 31, 39, 43, 45]. Additionally, five intervention arms evaluated the effect of the SMT program on power [13, 32, 33, 37, 45]. Seven intervention arms investigated the effect of the SMT program on speed [13, 15, 32, 33, 37, 39, 45]. Speed measurement is essential for evaluating athletic performance and its connection to ACL injuries, as high-speed activities can heighten injury risk, especially when combined with poor biomechanics and fatigue.

Additionally, four intervention arms examined the effect of the SMT program on core endurance [15, 36, 37, 41]. Core endurance tests assess stability by measuring how long a person can maintain a specific position. Furthermore, five intervention arms explored the effect of the SMT program on dynamic balance [15, 32, 36, 37, 40]. Dynamic balance was assessed using the Y Balance Test (YBT), which requires athletes to balance on one leg while reaching as far as possible with the other leg in three directions: anterior, posterolateral, and posteromedial.

Moreover, six intervention arms assessed the effect of the SMT program on knee valgus [15, 33–37]. Additionally, eight intervention arms examined the effect of the SMT program on landing errors [29, 34, 35, 40–42]. Landing errors were assessed using the Landing Error Scoring System (LESS) and Tuck Jump tests, both of which are functional assessments that evaluate an athlete’s landing mechanics, stability, and overall movement quality. Finally, four intervention arms investigated the effect of the SMT program on hamstrings/quadriceps strength and the hamstrings/quadriceps strength ratio [28, 30, 38, 44].

### Meta-analysis

#### Effect of SMT program on agility

**Fig. 2:**
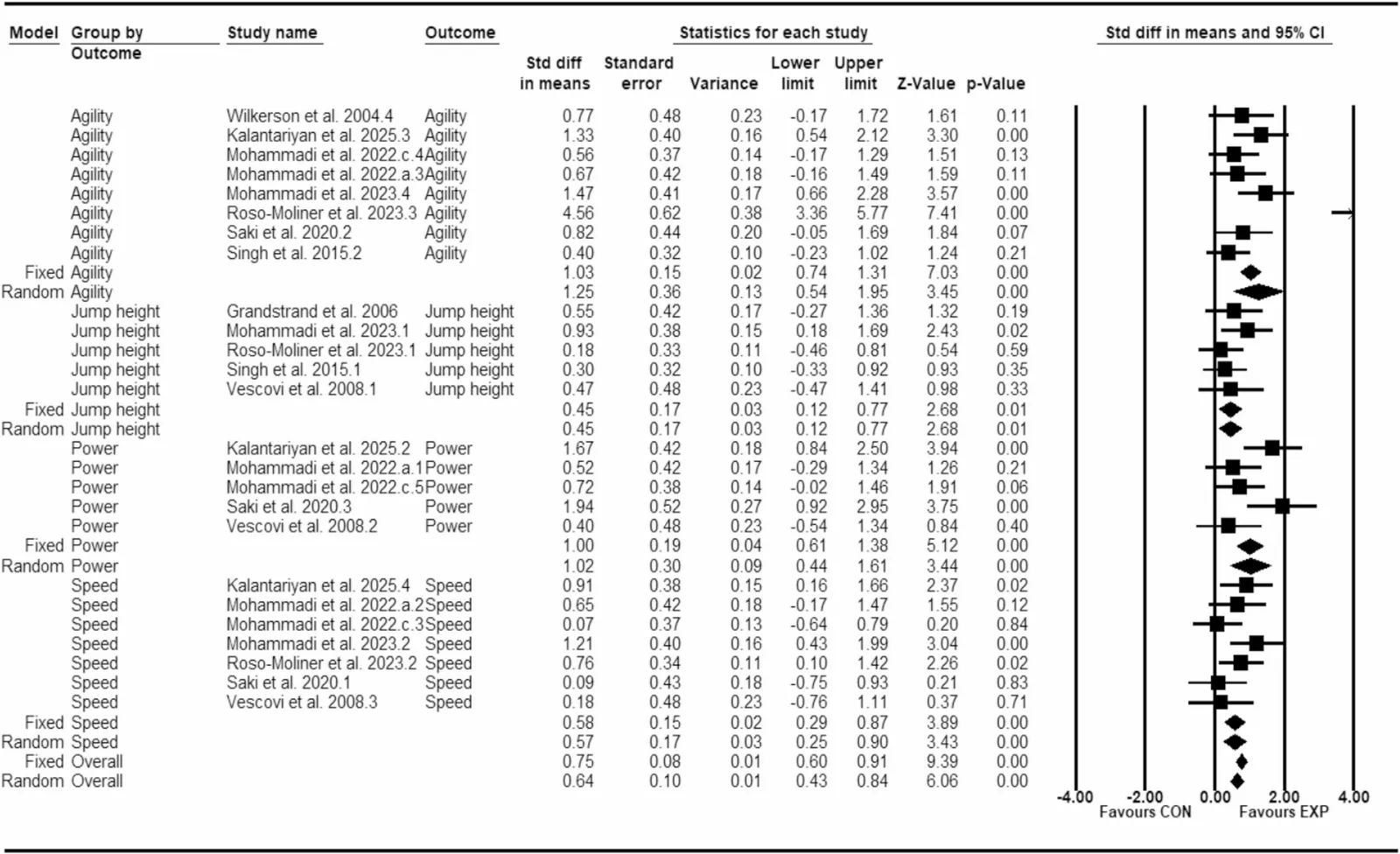
Forest plot of the SportsMetrics training program versus control group analysis on agility, jump height, power, and speed

The analysis included eight intervention arms examining the effect of the SMT program on the agility. Overall, SMT program demonstrated a significant improvement in the agility (1.24 [95% CI: 0.53–1.95], *p* = 0.001), and high significant heterogeneity among the studies (I² = 83.08%, *p* = 0.001); therefore, a random effects model was used (see Fig. 2). Both the visual assessment of funnel plots and the results from Egger’s test did not indicate any significant publication bias (*p* = 0.80). The trim-and-fill method identified two missing studies on the right side of the mean; however, the results remained unchanged after correction (1.34 [95% CI: 1.10–1.59]).

#### Effect of SMT program on jump height

The analysis included five intervention arms examining the effect of the SMT program on the jump height. Overall, SMT program demonstrated a significant improvement in the jump height (0.44 [95% CI: 0.12–0.77], *p* = 0.007), and no significant heterogeneity among the studies (I² = 00.00%, *p* = 0.62); therefore, a fixed effects model was used (see Fig. 2). Both the visual assessment of funnel plots and the results from Egger’s test did not indicate any significant publication bias (*p* = 0.80).

#### Effect of SMT program on power

The analysis included five intervention arms examining the effect of the SMT program on the power. Overall, SMT program demonstrated a significant improvement in the power (1.02 [95% CI: 0.44–1.60], *p* = 0.001), and moderate significant heterogeneity among the studies (I² = 56.62%, *p* = 0.001); therefore, a random effects model was used (see Fig. 2). Both the visual assessment of funnel plots and the results from Egger’s test did not indicate any significant publication bias (*p* = 0.62). The trim-and-fill method identified one missing study on the left side of the mean; however, the results remained unchanged after correction (0.84 [95% CI: 0.22–1.44]).

#### Effect of SMT program on speed

The analysis included seven intervention arms examining the effect of the SMT program on the speed. Overall, SMT program demonstrated a significant improvement in the speed (0.57 [95% CI: 0.28–0.87], *p* = 0.001), and no significant heterogeneity among the studies (I² = 20.17%, *p* = 0.27); therefore, a fixed effects model was used (see Fig. 2). Both the visual assessment of funnel plots and the results from Egger’s test did not indicate any significant publication bias (*p* = 0.52).

#### Effect of SMT program on core endurance

**Fig. 3:**
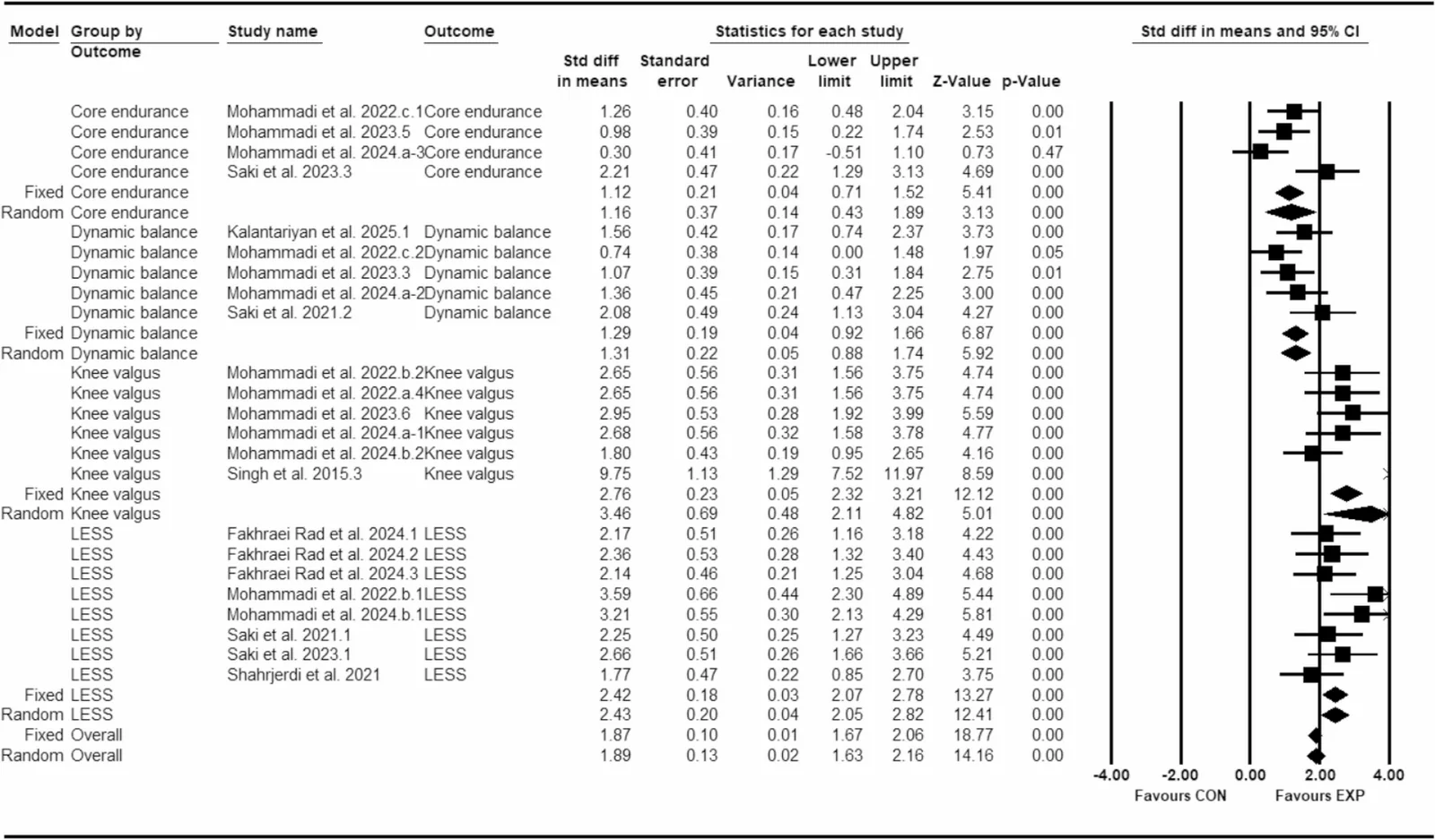
Forest plot of the SportsMetrics training program versus control group analysis on core endurance, dynamic balance, knee valgus, and landing error

The analysis included four intervention arms examining the effect of the SMT program on the core endurance. Overall, SMT program demonstrated a significant improvement in the core endurance (1.16 [95% CI: 0.43–1.89], *p* = 0.001), and moderate significant heterogeneity among the studies (I² = 68.77%, *p* = 0.02); therefore, a random effects model was used (see Fig. 3). Both the visual assessment of funnel plots and the results from Egger’s test did not indicate any significant publication bias (*p* = 0.30). The trim-and-fill method identified one missing study on the left side of the mean; however, the results remained unchanged after correction (0.85 [95% CI: 0.03–1.67]).

#### Effect of SMT program on dynamic balance

The analysis included five intervention arms examining the effect of the SMT program on the dynamic balance. Overall, SMT program demonstrated a significant improvement in the dynamic balance (1.29 [95% CI: 0.92–1.66], *p* = 0.001), and no significant heterogeneity among the studies (I² = 27.17%, *p* = 0.24); therefore, a fixed effects model was used (see Fig. 3). Both the visual assessment of funnel plots and the results from Egger’s test did not indicate any significant publication bias (*p* = 0.60). The trim-and-fill method identified one missing study on the left side of the mean; however, the results remained unchanged after correction (1.15 [95% CI: 0.68–1.64]).

#### Effect of SMT program on knee valgus

The analysis included six intervention arms examining the effect of the SMT program on the knee valgus. Overall, SMT program demonstrated a significant reduction in the knee valgus (3.46 [95% CI: 2.10–4.81], *p* = 0.001), and high significant heterogeneity among the studies (I² = 88.39%, *p* = 0.001); therefore, a random effects model was used (see Fig. 3). Both the visual assessment of funnel plots and the results from Egger’s test did not indicate any significant publication bias (*p* = 0.13).

#### Effect of SMT program on landing error

The analysis included eight intervention arms examining the effect of the SMT program on the landing error. Overall, SMT program demonstrated a significant reduction in the landing errors (2.45 [95% CI: 2.06–2.78], *p* = 0.001), and no significant heterogeneity among the studies (I² = 12.82%, *p* = 0.33); therefore, a fixed effects model was used (see Fig. 3). Both the visual assessment of funnel plots and the results from Egger’s test did not indicate any significant publication bias (*p* = 0.11).

#### Effect of SMT program on hamstrings strength

**Fig. 4:**
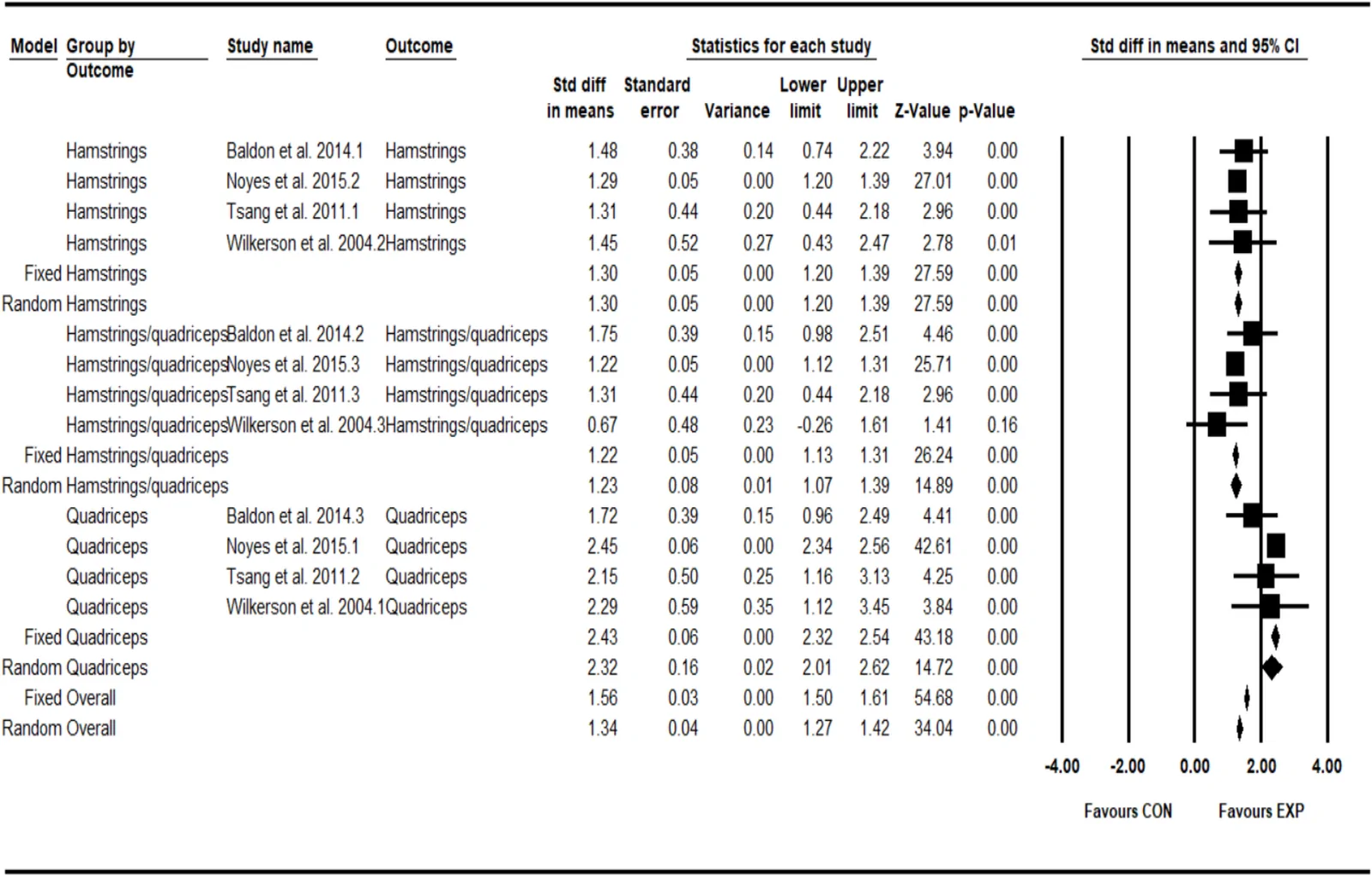
Forest plot of the SportsMetrics training program versus control group analysis on hamstrings/quadriceps strength

The analysis included four intervention arms examining the effect of the SMT program on the hamstring strength. Overall, SMT program demonstrated a significant improvement in the hamstring strength (1.29 [95% CI: 1.20–1.38], *p* = 0.001), and no significant heterogeneity among the studies (I² = 00.00%, *p* = 0.95); therefore, a fixed effects model was used (see Fig. 4).

#### Effect of SMT program on quadriceps strength

The analysis included four intervention arms examining the effect of the SMT program on the quadriceps strength. Overall, SMT program demonstrated a significant improvement in the quadriceps strength (2.43 [95% CI: 2.32–2.54], *p* = 0.001), and no significant heterogeneity among the studies (I² = 21.29%, *p* = 0.28); therefore, a fixed effects model was used (see Fig. 4).

#### Effect of SMT program on hamstrings/quadriceps strength ratio

The analysis included four intervention arms examining the effect of the SMT program on the hamstrings/quadriceps strength ratio. Overall, SMT program demonstrated a significant improvement in the hamstrings/quadriceps strength ratio (1.22 [95% CI: 1.13–1.31], *p* = 0.001), and no significant heterogeneity among the studies (I² = 5.27%, *p* = 0.36); therefore, a fixed effects model was used (see Fig. 4). Both the visual assessment of funnel plots and the results from Egger’s test did not indicate any significant publication bias (*p* = 0.86).

## Discussion

The present systematic review and meta-analysis aimed to evaluate the effect of SMT program on functional performance, landing error, dynamic knee valgus and hamstrings/quadriceps strength in athletes. The findings indicate that SMT significantly and positively improved functional performance, increased hamstrings/quadriceps strength, and reduced landing errors and dynamic knee valgus in athletes. To our knowledge, there has not been any study or comprehensive meta-analysis examining the extent to which SMT influences the functional performance, landing error, dynamic knee valgus and hamstrings/quadriceps strength of athletes. Other meta-analyses [19–23] assessing the effectiveness of NM training programs in reducing ACL injury rates have determined that these interventions are typically beneficial.

The findings of this review indicate that original research investigating the impact of SMT on athletes has predominantly concentrated on identifying risk factors associated with ACL injuries. Although these studies aimed to modify injury rates among athletes participating in specific sports, such as football, basketball, and volleyball, no original study has prospectively evaluated the direct effects of SMT on injury rates. Consequently, it appears essential for future original research to prioritize the examination of SMT’s influence on the injury rates of athletes. Moreover, the studies employed measurement tools with standardized units to assess the outcomes, facilitating distinct meta-analyses for each result.

Furthermore, Egger’s test was utilized to assess publication bias, indicating that there was no bias present in the research findings. However, two of the eight outcomes examined in this systematic review exhibited notable methodological shortcomings and variability across several dimensions. These dimensions included assessment tools and techniques, type of sport, volume and intensity of training, oversight by therapists or trainers, study populations, duration of sessions, characteristics of the training regimen, and overall length of the training programs. While the variability in assessment tools was mitigated by categorizing the variables, other limitations contributed to the increased heterogeneity among the studies. Notably, age range alone cannot account for this variability, as the age of study participants did not differ significantly across the included studies. Instead, methodological differences, such as variations in training protocols and the specific sports involved, likely played a more critical role in shaping the results. The demands of different sports may lead to distinct adaptations in agility and knee mechanics, while the specific measurement techniques employed could yield varying outcomes. Furthermore, the baseline fitness levels and prior training experiences of participants may further contribute to the differences observed. Therefore, it is advisable for future research in this area to consider these factors to draw more precise conclusions about the effects of SMT programs on athletes.

The results demonstrated positive effects of SMT on agility, jump height, power, and speed. SMT programs that incorporate strength, power, and plyometric exercises can effectively enhance acceleration and short-distance speed performance through consistent training methods. Improvements in the power of the hip, knee, and ankle extensors have been linked to enhanced speed ability [50], and selecting exercises that emphasize horizontal stimuli is likely to facilitate adaptations related to acceleration performance [51]. Therefore, integrating a diverse range of exercises that promote explosive power and speed, rather than concentrating solely on exercises with added resistance, may prove to be a more effective strategy for improving vertical jump performance in female athletes [52]. Improvements in agility are linked to enhanced neuromuscular control and the ability to respond quickly to dynamic movements, which are often associated with sports and physical activities. Enhanced agility allows athletes to change direction rapidly while maintaining balance, reducing the likelihood of awkward landings or falls that can lead to injuries. By training agility, athletes may also improve their proprioceptive abilities, which contribute to better joint stability and alignment during high-intensity activities [53]. Power is a critical physiological attribute defined as the product of strength and speed. This definition underscores the importance of both components in achieving optimal performance in various athletic and functional activities. Strength refers to the maximum force that a muscle or group of muscles can exert, while speed pertains to the rapidity with which this force can be applied. Together, these elements form the foundation for explosive movements, essential in sports and physical activities [54]. To enhance power effectively, training strategies must target both strength and speed components. A fundamental principle for improving lower limb muscle power involves focusing on strength exercises designed to induce muscle hypertrophy. Hypertrophy increases the cross-sectional area of muscle fibers, thereby augmenting the muscle’s capacity to generate force [55]. Concurrently, plyometric exercises play a vital role in enhancing muscle performance by promoting rapid muscle shortening during explosive movements such as jumps. These exercises not only improve the rate of force development but also contribute to increased muscle strength capacity, creating a synergistic effect when combined with traditional strength training [54]. Research indicates that the integration of plyometric and strength training yields superior improvements in muscle strength and power compared to isolated training modalities. This enhanced effect is attributed to the activation of fast-twitch muscle fibers and the nervous system priming that occurs during plyometric activities. Furthermore, the sequencing of these exercises is crucial; executing plyometric exercises prior to strength training has been shown to maximize explosive power [55]. This approach allows athletes to capitalize on the pre-activation of muscle fibers, leading to greater force production during subsequent strength exercises [54]. In the context of strength and conditioning programs, the strategic incorporation of plyometric exercises before strength training can significantly enhance overall program effectiveness. This sequencing not only optimizes power output but also contributes to improved athletic performance across various sports disciplines [55]. By understanding and applying the principles of power training, athletes can achieve greater levels of performance and efficiency in their respective fields.

Furthermore, the results showed positive impacts of SMT on core endurance. Part of the SMT involves NM training focused on the core region, specifically targeting the abdominal muscles and the muscles of the lower limbs and thighs [41]. This highlights the effectiveness of the SMT in enhancing core stability. Weak core stability can negatively affect the alignment of the lower limbs during dynamic movements [56], leading to disrupted movement patterns and improper execution, which can ultimately reduce neuromuscular coordination in the lower limbs, particularly during landing tasks [57]. Therefore, significant emphasis is placed on strengthening the core and improving lower limb performance to address functional weaknesses through specialized injury prevention exercises [58]. Since SMT is fundamentally designed for injury prevention and performance enhancement, incorporating core stability and lower limb-specific neuromuscular programs, it is highly recommended for athletes and coaches to utilize this training to help prevent serious injuries, including ACL tears.

Also, the results showed positive impacts of SMT on dynamic balance. The SMT program encompasses a dynamic warm-up, plyometric jump training, and strengthening exercises targeting the lower and upper body as well as the core, with a significant emphasis on balance. Balance is maintained through two mechanisms: static stability, which is supported by joint capsules, ligaments, and bones, and dynamic stability, which relies on muscle performance. The lateral ligaments of the knee provide secondary and reflexive protection to the joint [59]. A proper warm-up enhances the sensitivity of mechanical receptors in the joints by increasing blood flow to active muscles and raising their oxygen levels. This process stimulates the tissues that support and protect the joints, enabling better control during dynamic movements and helping to restore stability and balance for athletes in challenging positions [60]. There is a clear connection between balance and muscle strength, as studies have shown that both strength training and plyometric exercises can improve athletes’ balance [61].

Additionally, the results indicated a significant reduction in knee valgus due to SMT. Additionally, demonstrated a significant decrease in landing errors as a result of SMT. Excessive lateral trunk flexion and insufficient neuromuscular control can shift the center of gravity over the knee joint, resulting in knee valgus [62]. Additionally, weakness in the thigh muscles, particularly the lateral muscles, contributes to an increased angle of knee valgus [63]. NM training programs, such as SMT, can effectively reduce landing forces, knee abduction and adduction range of motion, and dynamic knee valgus [12]. While the emphasis on knee valgus is important, it is indeed essential to recognize that knee alignment does not exist in isolation. Adjustments at the hip and ankle can significantly influence overall biomechanics and may compensate for suboptimal knee positioning [64]. Additionally, ground reaction forces (GRFs) are crucial in determining the impact of landing mechanics on injury risk; they can alter the stress distribution across the knee and surrounding joints during dynamic activities [65]. To mitigate the risk of knee injuries, it is essential to reduce the impact transmitted through the lower extremity joints, thereby facilitating softer landings [64]. Several studies have assessed GRFs and angular impulses in the lower extremity joints to analyze landing biomechanics [64, 65]. Tamura, Akasaka and Otsudo (66) found that soft landings characterized by a knee flexion angle exceeding 90 degrees after descending from a height of 59 cm produced lower GRFs and ankle plantar flexor impulses compared to stiff landings. Consequently, soft landings effectively decrease the impact forces exerted on the lower extremity joints during the ground contact phase [66]. These findings suggest that kinetic variables such as GRFs and angular impulses are valuable metrics for evaluating the quality of landings. Additionally, recent studies indicate that landing with knee valgus alignment may lead to suboptimal dynamic lower extremity alignment, termed dynamic knee valgus, which results from a combination of hip adduction, hip internal rotation, and knee abduction, consequently increasing the knee valgus angle or the knee abduction moment [62, 67]. Therefore, a holistic perspective that encompasses multi-joint interactions, functional muscle coordination, and GRFs would enhance our understanding of how SMT can effectively contribute to ACL injury prevention. By integrating these factors, researchers and practitioners can develop more effective training interventions that address the multifaceted nature of knee injuries.

The components of the SMT program, particularly plyometrics and a focus on landing techniques, are specifically designed to modify these kinetic variables, which in turn contribute to the observed reductions in landing errors and knee valgus. Plyometric exercises, which emphasize explosive movements, enhance the athletes’ ability to generate force rapidly and improve their neuromuscular control during dynamic activities. This increased strength and power enable athletes to better manage GRFs during landings, reducing the impact forces transmitted through the lower extremities. Additionally, the focused instruction on proper landing techniques encourages athletes to adopt more biomechanically sound postures, such as maintaining alignment of the knees over the toes and engaging the hips and core during landings. This technique not only minimizes the risk of excessive knee valgus but also promotes effective energy absorption, thereby reducing the likelihood of landing errors. By systematically addressing these kinetic variables through targeted training components, the SMT program effectively enhances athletes’ movement mechanics, leading to improved performance outcomes and a lower incidence of injuries associated with poor landing strategies. Thus, the integration of plyometrics and landing technique focus within the SMT program is crucial for modifying GRFs and optimizing athletic performance.

Optimal muscle strength in the core and lower extremities is crucial for protecting the knee joint. Beyond adequate core strength, dynamic stability and motor control are essential for improving landing test scores [40]. The quadriceps and hamstrings are key muscles for knee extension and flexion movements. An imbalance between these muscle groups increases the risk of knee joint injury, often linked to a neuromuscular dysfunction characterized by quadriceps dominance [68]. When the ability to activate the quadriceps exceeds that of the hamstrings, it can lead to knee extension during landing, a factor associated with ACL tears. Thus, strategies focusing on activating the quadriceps while promoting knee flexion through the hamstrings are vital for improving landing test performance [69]. The findings suggest that SMT training effectively fosters a functional muscular balance between the quadriceps and hamstrings during training sessions.

Moreover, the results showed positive impacts of SMT on hamstring strength, quadriceps strength, and hamstrings/quadriceps strength ratio. Strength imbalances, particularly the quadriceps to hamstrings (Q: H) ratio, are significant risk factors for knee injuries in both male and female athletes. Research indicates that female athletes are at a higher risk of ACL injuries due to a poor Q: H ratio, often characterized as “quadriceps dominant [70].” Studies have shown that, in response to anterior tibial translation perturbations, female athletes tend to activate their quadriceps before their hamstrings, unlike male athletes [71, 72]. Additionally, female athletes often adopt an upright posture with extended knees and hips when landing from jumps [71]. This combination of factors increases stress on the ACL, leading to a higher incidence of injuries in women [70]. The SMT program implemented in this systematic review successfully improved both hamstring and quadriceps strength, with a more significant impact on hamstring strength. Although the study did not specifically examine the relationship, the increase in hamstring strength without a corresponding rise in quadriceps strength suggests a potentially effective way to enhance the Q: H ratio, which merits further research. During SMT training sessions, the focus was on proper technique, overall posture, and correct take-off and landing positions [28]. This emphasis likely contributed to the observed increases in hamstring strength, as the exercises involved stretching the hamstrings during both preparatory and landing phases. The hamstrings play a vital role during jumping and landing by providing stabilization through eccentric contraction, which helps counteract the forces exerted by the quadriceps [71]. Eccentric muscle actions can generate greater maximum forces compared to concentric or isometric actions, making them particularly effective for building overall strength [44].

The large effect sizes observed for dynamic knee valgus (SMD = 3.46) and quadriceps strength (SMD = 2.43) in the context of the SMT program can be justified by several factors. Firstly, the program is specifically designed to enhance neuromuscular control and strength, targeting key muscle groups involved in lower extremity biomechanics. Athletes participating in the program, particularly those who may have been untrained or less experienced, are likely to exhibit significant improvements due to their initial baseline deficiencies, leading to a greater magnitude of change. Additionally, the systematic review and meta-analysis included studies that utilized rigorous training protocols and specific measurement tools that may have been particularly effective in capturing these improvements. The high variability in responses among untrained participants, combined with the focused nature of the intervention, likely contributed to the inflated effect sizes observed for both dynamic knee valgus and quadriceps strength, underscoring the program’s efficacy in addressing these critical performance metrics.

### Limitations and future scope

This study presents several methodological limitations that warrant further investigation: (1) None of the included studies implemented allocation concealment, indicating a significant risk of selection bias. This absence raises concerns about the integrity of the randomization process, as it may have influenced the assignment of participants to interventions, potentially skewing the results. (2) The follow-up durations in the included studies were generally short. This limitation restricts our ability to assess the long-term effects and sustainability of the interventions. Short follow-up periods may not capture delayed outcomes or the potential for relapse, thereby affecting the overall conclusions drawn from the findings. Future research should focus on addressing the limitations identified in this study by proposing more specific and robust methodologies. We recommend conducting multicenter, long-term, large-sample prospective RCTs to directly investigate the incidence of ACL injuries. Such studies would provide a more comprehensive understanding of the factors contributing to ACL injuries across diverse populations and settings. Additionally, these prospective RCTs should aim to assess the sustainability of training benefits over extended periods. By evaluating the long-term effects of various intervention strategies, researchers can determine which approaches are most effective in preventing ACL injuries and maintaining physical performance. 4) Another important consideration is the potential for issues with intervention adherence among participants. Variability in adherence can lead to inconsistencies in the effectiveness of the interventions, making it challenging to determine their true impact. Future studies should incorporate strategies to monitor and enhance adherence to ensure more reliable results. Furthermore, incorporating standardized measures for intervention adherence and follow-up assessments will enhance the reliability of the findings. 5) While the research conducted in this study exhibited a high-fair level of methodological rigor, indicating that the findings of the current meta-analysis are both robust and trustworthy, considerable heterogeneity in some outcomes necessitates caution when interpreting the results and generalizing the findings to all training types. 6) Notable publication bias was observed in several analyses. To mitigate this, the authors applied the trim-and-fill correction method, suggesting that the meta-analysis results were not significantly influenced by the observed publication bias. However, the limitations of the random-effects model in the context of heterogeneity resulted in a greater emphasis on smaller studies, and the funnel plot asymmetry distorted the results towards those smaller studies. 7) Also, one notable limitation of our literature search is the exclusion of the EMBASE database, which was inaccessible due to an embargo. This restriction may have limited our ability to identify certain studies that are typically indexed in EMBASE, potentially affecting the comprehensiveness of our review. However, we mitigated this limitation by conducting a thorough manual search in Google Scholar, which allowed us to capture a wide range of relevant literature. Given the extensive nature of our manual search and the breadth of studies available through Google Scholar, we believe that the likelihood of missing high-quality studies is low. Nonetheless, we acknowledge that the absence of EMBASE could have resulted in the omission of some pertinent research, and we recommend that future studies consider including this database to enhance the robustness of their literature reviews. 8) The language scope of the search in this systematic review was restricted to English, indicating a need for future studies to encompass a broader range of languages to enhance the comprehensiveness of the findings.

## Conclusion

In conclusion, the present systematic review and meta-analysis demonstrate that the SMT program significantly and positively improved functional performance, increased hamstrings/quadriceps strength, and reduced landing errors and dynamic knee valgus in athletes. Despite the robust evidence, there remains a gap in original research directly linking SMT to injury rates, underscoring the need for future studies to explore this relationship.

## Supplementary Information

Supplementary Material 1.

Supplementary Material 2.

Supplementary Material 3.

## Data Availability

The datasets used and/or analyzed during the current study are available from the corresponding author on reasonable request.

## Abbreviations

ACL: Anterior cruciate ligament
SMT: SportsMetrics training
CT: Conventional training
NM: Neuromuscular
