# Supplementary material for: The effect of SportsMetrics training program on functional performance, landing error, dynamic knee valgus and hamstrings/quadriceps strength in athletes: a systematic review and meta-analysis
Source: BMC Sports Sci Med Rehabil. 2026 Aug 14;18:583. doi: 10.1186/s13102-026-01983-4 (PMC13757088; doi:10.1186/s13102-026-01983-4)
Supplement: Supplementary file 3 [file 13102_2026_1983_MOESM3_ESM.docx]

**Supplementary file 3.** Quality of the evidence using the GRADE system

| **Quality assessment** | | | | | | **Quality**  **of evidence** |
| --- | --- | --- | --- | --- | --- | --- |
| **Outcomes** | **Risk of bias** | **Inconsistency** | **Indirectness** | **Imprecision** | **Publication Bias** |  |
| Agility | No serious limitation | Serious limitation | No serious limitation | No serious limitation | No serious limitation |  |
| Jump height | No serious limitation | No serious limitation | No serious limitation | No serious limitation | No serious limitation |  |
| Power | No serious limitation | Serious limitation | No serious limitation | No serious limitation | No serious limitation |  |
| Speed | No serious limitation | No serious limitation | No serious limitation | No serious limitation | No serious limitation |  |
| Core endurance | No serious limitation | Serious limitation | No serious limitation | No serious limitation | No serious limitation |  |
| Dynamic balance | No serious limitation | No serious limitation | No serious limitation | No serious limitation | No serious limitation |  |
| Knee valgus | No serious limitation | Serious limitation | No serious limitation | No serious limitation | No serious limitation |  |
| Landing error | No serious limitation | No serious limitation | No serious limitation | No serious limitation | No serious limitation |  |
| Hamstrings/quadriceps strength ratio | No serious limitation | No serious limitation | No serious limitation | No serious limitation | No serious limitation |  |
